# Effect of DNA sequence of Fab fragment on yield characteristics and cell growth of *E. coli*

**DOI:** 10.1038/s41598-017-03957-6

**Published:** 2017-06-19

**Authors:** Antti Kulmala, Tuomas Huovinen, Urpo Lamminmäki

**Affiliations:** 1 Department of Biochemistry/Biotechnology, University of Turku, 20520 Turku, Finland; 2 Department of Biochemistry, University of Cambridge, Cambridge, CB2 1GA UK

## Abstract

Codon usage is one of the factors influencing recombinant protein expression. We were interested in the codon usage of an antibody Fab fragment gene exhibiting extreme toxicity in the *E. coli* host. The toxic synthetic human Fab gene contained domains optimized by the “one amino acid-one codon” method. We redesigned five segments of the Fab gene with a “codon harmonization” method described by Angov *et al*. and studied the effects of these changes on cell viability, Fab yield and display on filamentous phage using different vectors and bacterial strains. The harmonization considerably reduced toxicity, increased Fab expression from negligible levels to 10 mg/l, and restored the display on phage. Testing the impact of the individual redesigned segments revealed that the most significant effects were conferred by changes in the constant domain of the light chain. For some of the Fab gene variants, we also observed striking differences in protein yields when cloned from a chloramphenicol resistant vector into an identical vector, except with ampicillin resistance. In conclusion, our results show that the expression of a heterodimeric secretory protein can be improved by harmonizing selected DNA segments by synonymous codons and reveal additional complexity involved in heterologous protein expression.

## Introduction

Antibody fragments are increasingly used for a variety of applications in diagnostics, basic life science research, and as therapeutics^1^. There are several advantages to fragmented antibodies over the full-length IgGs, including better penetration into tissues and faster clearance for imaging purposes. The lack of Fc domain reduces toxic effects in therapy and target-independent interference in IVD assays^2–4^. Furthermore, the small size of antibody fragments makes them more amenable to genetic modifications (e.g. for fusion proteins and site-specific chemical tagging)^5^. Another useful property of antibody fragments is their easy expression in *E. coli*, which enables easier production with lower costs, and compatibility with phage display^4, 6^.

The most widely used antibody fragments are single-chain variable fragment (ScFv) and antigen binding fragment (Fab)^7^. Fab, which is a hetero-dimer of the light chain and the two N-terminal domains of the heavy chain (V_H_ and C_H1_), is the preferred choice in many applications. It generally shows better stability compared to the ScFvs, and can more easily be detected with different secondary antibodies. In addition, due to the structural similarity, the Fabs can be converted to full-length IgGs, or vice versa, with a greater chance of retaining functionality than ScFvs^8, 9^.

Unfortunately, the yields of Fab fragments in *E. coli* expression often are relatively low, which causes difficulties for scale-up and purification, increasing the production costs^1, 10^. The low expression levels of Fab fragments have been attributed to toxicity effects, intracellular degradation, aggregation and inefficient translocation^11, 12^. To alleviate these problems different vector elements, bacterial strains, and cultivation conditions can be tested^7, 13^. Indeed, Fab expression has been studied by comparing the effect of different promoters in combination with different *E. coli* strains^10^ and by altering culture media and aeriation conditions^1^. In addition, the influence of altered light and heavy chain gene order, temperature and chaperone co-expressions have been explored^11, 14, 15^.

It is currently known that codon usage can affect protein expression, for example, by altering protein folding^16–18^ and by modulating the efficiency of translation initiation^19^. Studies with bacteria have suggested that the efficiency of translation initiation is inversely correlated to the stability of mRNA secondary structures near the 5′ end^20–22^. Synonymous codon alteration at the signal sequence can also significantly affect protein yield^23^ by influencing the folding of the mature protein^24^ or by facilitating the binding of the signal recognition particle (SRP) consequently leading to more efficient translocation^25^. Although two studies have shown that codon usage can also affect the properties^26^ and yield^27^ of ScFv antibodies in *E. coli*, codon usage has not obtained much attention as a solution to the Fab expression problems. In Fab fragments, the synonymous encoding may have additional effects due to the heterodimeric nature of the molecule, for example, by affecting the translation stoichiometry of the two chains^28^. In multi-domain proteins, such as Fab, the translation speed of the succeeding domains may also be a crucial parameter for successful expression^29^.

The starting point of this study was a synthetic human Fab fragment (named sFab) that contained domains optimized by the “one amino acid-one codon” method. A surprising lethal effect was observed upon transformation into *E. coli* of the codon optimized expression construct. In order to alleviate the toxic effects on the host and to restore protein production, several DNA segments of the sFab gene were altered by “codon harmonization” method described by Angov *et al*.^16^. We investigated the contribution of the individual redesigned gene segments on cell viability, Fab expression and phage display efficiency. We also observed that chloramphenicol and ampicillin resistance gene markers exerted differential effects on cell viability and protein production depending on the specific DNA sequences of the synonymously coded Fab fragments.

## Materials and Methods

### Vectors, bacterial strains, enzymes, reagents and sequencing

Phagemid vector pEB32x was used for phage display^30^ and vectors pAK400^31^ and pLK04^32^ for soluble expression. Vector pUC57 was obtained from Genscript (Piscataway Township, USA). *E. coli* XL-1 Blue (*recA1 endA1 gryA96 thi-1 hsdR17 supE44 relA1 lac* [*F*’ *proAB lacI* ^*q*^ *ZΔM15 Tn10* (*Tet* ^*r*^)]) was purchased from Stratagene (LaJolla, USA) and BL21 (*B F* ^−^ *ompT gal dcm lon hsdS*_*B*_(*r*_*B*_^−^ *m*_*B*_^−^) [*malB* ^+^]_*K*−*12*_(*λ* ^*S*^)) was purchased from Merck (Darmstadt, Germany). Minipreps, gel extractions and PCR purifications were done with Thermo Scientific kits (Waltham, USA). All enzymes and enzyme buffers were obtained from New England Biolabs (Ipswich, USA) and used according to the manufacturer’s instructions unless otherwise noted. The parental Fab fragment genes Fab0 and sFab were purchased from Genscript and Entelechon, respectively. They composed of a human gammaglobulin subtype 1 heavy chain and subtype kappa light chain (Supplementary Fig. 1). All DNA sequencing was implemented at Macrogen Inc. (Seoul, South Korea).

### Statistical analysis

All statistical analyses were performed with IBM SPSS Statistics 22 (Armonk, USA). The Fab variants were compared to the parent Fab0 in a pairwise fashion. Since the sampling sizes were small Mann-Whitney U test was used for comparing generation times and the total yields of immunoreactive Fab. Spearman’s rank correlation was used for the analysis of the relationship between mRNA folding stability and the total Fab yield as it is less sensitive to outliers than Pearson correlation and does not assume normal distribution of the dependent variables. Cut-off value for significance was *p* ≤ 0.05 for all the tests.

### Analysis of the mRNA minimum free energy

The minimum free energy of secondary structure formed by the mRNA of each variant and parent gene were predicted with NUPACK web software^33^. The whole coding sequence of the bicistronic mRNA (1449 bp, including 27 bp non-coding region between the C_K_ and the heavy chain PelB signal sequence) was analyzed at the applied 26 °C induction temperature.

### tRNA adaptation index (tAI)

Analysis of the codon usage was performed by using tRNA adaptation index (tAI) described by dos Reis *et al*.^34^. The tAI index indicates, how well the gene has been adapted to the genomic tRNA pool. The tAI index is calculated by using the relative adaptiveness value (*w*_*i*_), which is assigned for each codon. Before relative adaptiveness value can be calculated, absolute adaptiveness value (*W*_*i*_) for each codon (*i*) has to be calculated. Absolute adaptiveness (*W*_*i*_) is calculated as described by eq. (1) where *n*_*i*_ is the number of tRNA isoacceptors that recognize the *i*th codon, *tGCN*_ij_ is the gene copy number of the *j*th tRNA that recognize the *i*th codon. *S*_*ij*_ is the selective constraint on the efficiency of codon-anticodon coupling.

1 $${W}_{i}=\sum _{j=1}^{{n}_{i}}(1-{S}_{ij})tGC{N}_{ij}$$

The relative adaptiveness value (*w*_i_) can be calculated by dividing the absolute adaptiveness value (*W*_*i*_) of each codon by the maximal *W*_*i*_ value (*W*_*i*_/*W*_*max*_). The tAI index is simply a geometric mean of the relative adaptiveness values and can be calculated as described by eq. (2) where *i*_*kg*_ is the codon defined by *k*_*th*_ triplet in a gene *g* and *l*_*g*_ is the length of gene in codons.

2 $$tA{I}_{g}={(\prod _{k=1}^{{l}_{g}}{w}_{ikg})}^{1/{l}_{g}}$$

The relative adaptiveness values that were used to calculate tAI-values in this study were obtained from the table compiled by Tuller *et al*.^35^.

### Cloning of the genetic variants

Fab variants were produced by replacing DNA segments of the functional parent gene (Fab0) with respective extracted DNA segment from the non-functional parent gene (sFab). For the production of variants 1, 2 and 3, parent gene Fab0 and parent gene sFab were first amplified by PCR. The parent genes were in pEB32x and pUC57 vectors, respectively. Amplified Fab0 gene was then digested with SfiI (Thermo Scientific) and subsequently further digested in three separate reactions with (1) SexAI, with (2) SexAI and MscI and with (3) MscI and PacI. After digestion, target fragments (1) 1273 bp^V1^, (2) 1094 bp^V2^ and 131 bp^V2^, (3) 734 bp^V3^ and 310 bp^V3^ were obtained by gel extraction. Amplified sFab gene was digested in the same manner as Fab0 and (1) 131 bp^V1^, (2) 179 bp^V2^ and (3) 360 bp^V3^ target fragments were extracted from the gel. The variants 1, 2 and 3 were then formed by mixing appropriate target fragments (all the fragments having the same superscript) and SfiI digested pEB32x vector together in equal molar ratios and ligating them with T4 DNA Ligase (Thermo Scientific) in three o/n reactions.

For the production of variants 4 and 5, the parent genes Fab0 and sFab in PEB32x and pUC57 vectors were digested with SfiI, PacI and XhoI in two separate reactions: (1) XhoI and SfiI and (2) XhoI and PacI. Target fragments (1) 885^V5^ bp and (2) 5486 bp^V4^ were obtained from Fab0 digestion. Target fragments (1) 519^V5^ bp and (2) 215^V4^ bp were obtained from sFab digestion. The variant 4 was formed by mixing the appropriate target fragments of Fab0 and sFab with T4 DNA Ligase in molar ratio of 1:5, respectively. Variant 5 was formed using “PCR-after-ligation” method^36^. The appropriate target fragments and SfiI digested pEB32x vector were first ligated with T4 DNA ligase in equal molar ratios. Ligation reaction was then amplified by PCR, purified with PCR purification kit and subsequently digested with SfiI. Reaction was purified with PCR purification kit and ligated to SfiI digested pEB32x vector with T4 DNA ligase. Molar ratio of pEB32x and digested PCR product was 1:3, respectively. All variants were transformed into *Escherichia coli* and plated as described by Huovinen *et al*.^37^. Minipreps were produced and validity of the variants was confirmed by sequencing. After confirmation of validity, all variants were subcloned into pLK04 and pAK400 vectors using T4 DNA ligase and SfiI sites. Molar ratio of vectors and inserts were 1:3. Again, the validity was confirmed by sequencing. More detailed description of cloning is provided in the Supplementary methods.

### Phage production

Effect of DNA sequence on phage production and phage immunoreactivity was studied by expressing all variants and both parent genes as a fusion to the p3 coat protein from phagemid vector. Phage production was carried out as described by ref. 38. Overnight pre-cultures were diluted to OD (600 nm) 0.05 in 20 ml of SB medium (1% glucose, 25 µg/ml cm, 10 µg/ml tet) and cells were cultured at 37 °C with 300 rpm shaking to OD (600 nm) 0.5. Cells were infected with VCS-M13 helper phage (Stratagene) with 20-fold multiplicity of infection and incubated at 37 °C for 30 min. After infection, cultures were centrifuged at 6800 g for 10 min at 4 °C. Supernatants were discarded and pellets were suspended in 20 ml of SB medium (0.05% glucose, 25 µg/ml cm, 10 µg/ml tet, 5 mM MgCl_2_). Cultures were grown at 26 °C for 1 h with 250 rpm shaking, after which kanamycin was added to 30 µg/ml final concentration and cultures were continued o/n. The following day, cultures were centrifuged at 6800 g for 10 min at 4 °C. Supernatants were collected and 1/6 volume of 20% PEG/1.5 M NaCl was added among supernatants to precipitate the phages. Precipitation reactions were incubated on ice for 1 h and subsequently centrifuged at 6800 g for 15 min at 4 °C. Phage pellets were dissolved in 1 ml of 1× TSA buffer (50 mM Tris-HCl, pH 7.5; 150 mM NaCl, 0.02% w/v NaN_3_) and subsequently centrifuged with tabletop centrifuge at 16 300 g, for 5 min at 4 °C. Supernatants were transferred to new tubes. Phage productions were repeated three times.

### Determination of phage titer by OCCA

Number of phage particles was determined by OCCA (oligonucleotide-directed chelate complementation assay) method^39^. Phage samples were first diluted 1/100 in 1× TBS buffer (50 mM Tris-HCl, pH 7.5; 150 mM NaCl) and 10 µl of the dilutions were mixed with 60 µl of reaction solution (25 mM Tris-HCl, pH 7.5; 10 nM Eu-oligonucleotide, 5 nM antenna oligonucleotide, 900 nM NaCl. 0.1% Tween-40, 0.05% NaN_3_, 30 µM DTPA). Samples were incubated at 95 °C for 1 min and subsequently cooled down to 25 °C. After incubation, samples were pipetted on a C-12 Low Fluor Maxi Strips Yellow 96 well plate (Nunc, Rochester, USA) as triplicate. Plate was incubated at RT for 15 min with slow shaking, after which time-resolved europium signal was measured with Victor 1420 Multilabel Counter (Wallac, Turku, Finland).

### Determination of phage immunoreactivity by immunoassay

Immunoreactivity of the produced phage particles was determined by phage immunoassay. Wells of a 96 well streptavidin plate (Kaivogen, Turku, Finland) were coated with 100 µl per well of 1 µM biotinylated digoxigenin in Assay Buffer (Kaivogen). The streptavidin plate was incubated at RT for 1 h with slow shaking. After incubation, the streptavidin plate was washed two times with Delfia Plate Wash (Wallac) by using Kaivogen wash buffer. Same amount of phages from each phage production (1 × 10^9^ pfu) were added on the wells in 100 µl of Assay Buffer as triplicate and incubated at RT for 1 h with slow shaking. After incubation, the streptavidin plate was washed two times. Digoxigenin bound phages were detected with 25 ng per 100 µl Eu-labeled mouse anti-phage antibody (University of Turku, Finland) by incubating at RT for 1 h with slow shaking. After incubation, the streptavidin plate was washed four times. Delfia Enhancement solution (Wallac) was added and the plate was incubated at RT for 10 min with slow shaking, after which time-resolved europium signal was measured with Victor 1420 Multilabel Counter.

### Protein production

The effect of DNA sequence on soluble Fab expression was studied by expressing all variants and both parental genes from vectors pAK400 and pLK04. The only difference between these vectors was the antibiotic resistance marker, chloramphenicol acetyltransferase (CAT) and ß-lactamase (TEM-1), respectively. Overnight pre-cultures were diluted to OD (600 nm) 0.1 in 20 ml of SB medium (1% glucose, 25 µg/ml cm or 100 µg/ml amp, 10 µg/ml tet) and cells were cultured at 37 °C with 300 rpm shaking to OD (600 nm) 0.7–0.9. After this, same amount of cells (2 × 10^9^ cells) were taken from each culture and centrifuged at 3220 g for 11 min at 20 °C. After centrifugation the supernatant was discarded and pellets dissolved in 5 ml of SB medium (1 mM IPTG, 10 µg/ml tet, 25 µg/ml cm or 100 µg/ml amp depending on the vector) to OD (600 nm) 0.5. Cells were cultured at 26 °C with 300 rpm shaking for 3 h. After induction, 1 ml sample was taken from each culture. Cells were pelleted with tabletop centrifuge at 16 300 g, for 5 min at 4 °C. Pelleted cells were suspended in Assay buffer and cells were disrupted by sonication while keeping on ice. Protein productions were repeated three times.

### Up-scaled protein production

In addition to small-scale protein productions, the harmonized parental gene Fab0 was expressed from pLK04 vector in BL21 strain in shake flask culture. Overnight pre-cultures were diluted to OD (600 nm) 0.1 in 2 × 200 ml of SB medium (1% glucose, 100 µg/ml amp). Ratio of culture volume to shake flask volume was 1:10. Cells were cultured at 37 °C with 300 rpm shaking to OD (600 nm) 0.7. Cells were pelleted by centrifugation at 3250 g for 11 min at 20 °C and supernatant was discarded. For the induction, cell pellets were re-suspended in 2 × 200 ml of SB medium (100 µg/ml amp, 1 mM IPTG). Induction was continued for 4 h at 26 °C with 300 rpm shaking. After induction, 1 ml sample was taken from the culture and it was treated the same way as in small-scale protein production. After sampling, cells were harvested by centrifugation at 8000 g for 10 min at +4 °C. Pelleted cells were re-suspended in 10 ml of 1× PBS (20 mM Na_2_HPO_4_, pH 7.4; 300 mM NaCl) and subsequently disrupted by sonication while keeping on ice. Cell debris was pelleted by centrifugation at 20 000 g for 20 min at +4 °C.

### Measurement of the yield of immunoreactive Fab fragment by immunoassay

The yields of immunoreactive Fab fragments were studied by immunoassays. Wells of a 96 well streptavidin plate were coated with 100 µl per well of 1 µM biotinylated digoxigenin in Assay Buffer. The streptavidin plate was incubated at RT for 1 h with slow shaking. After incubation, the streptavidin plate was washed two times with Delfia Plate Wash by using Kaivogen wash buffer. After washing, 100 µl per well of samples were added as triplicate. Samples were first diluted 1/100 in Assay Buffer before addition on the wells. For the quantification of immunoreactive Fab, 100 µl per well of purified Fab0 was used as a standard at final concentrations of 0; 0.0015; 0.003; 0.015; 0.03; 0.15; 0.3 µg/ml in Assay buffer. The streptavidin plate was incubated at RT for 1 h with slow shaking and subsequently washed two times with Delfia Plate Wash by using Kaivogen wash buffer. Immunoreactive Fabs were detected with 25 ng per 100 µl Eu-labeled anti-human Fab 2A11 (Hytest Ltd, Turku, Finland) and by incubating at RT for 1 h with slow shaking. The streptavidin plate was washed four times. Delfia Enhancement solution was added and the plate was incubated at RT for 10 min with slow shaking, after which time-resolved europium signal was measured with Victor 1420 Multilabel Counter.

## Results

### Characteristics of the parent genes and the genetic variants

**Figure 1:**
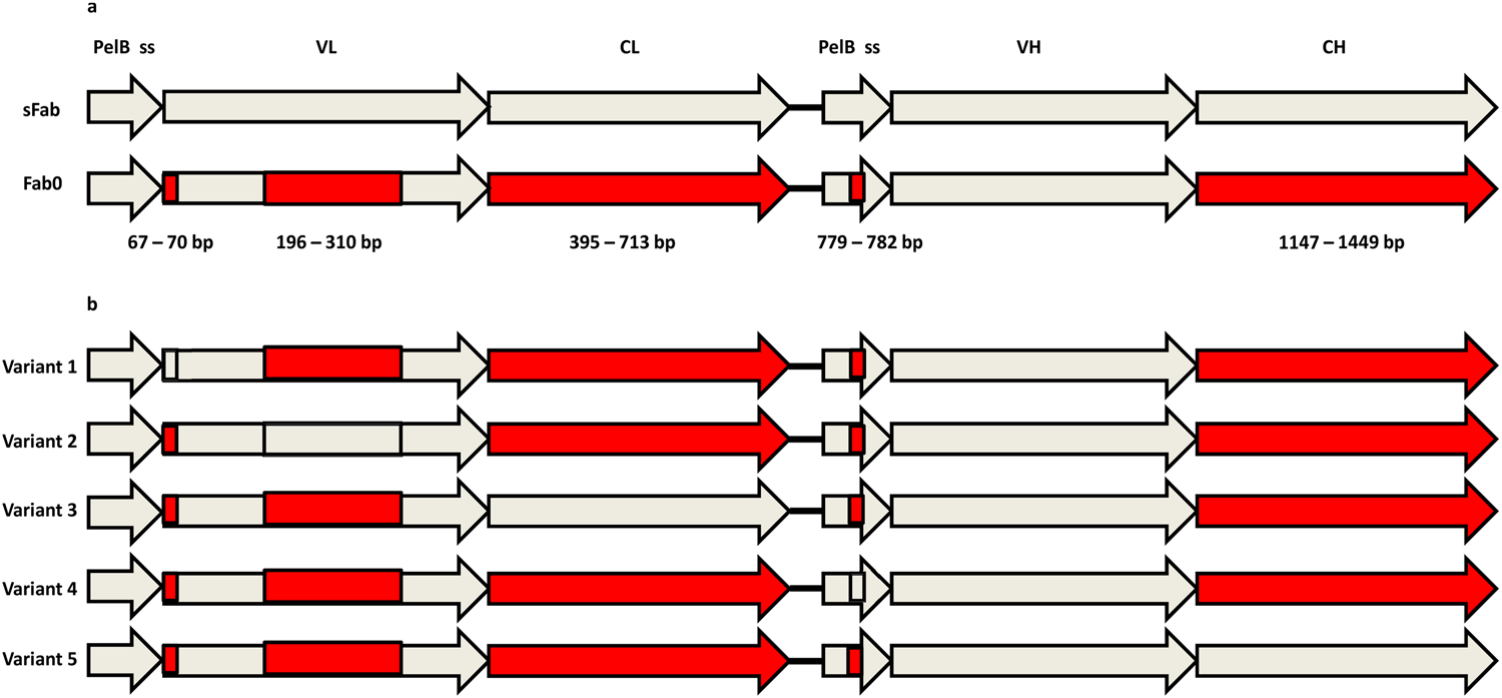
Illustration of the parent genes and the chimeric variants. (**a**) The parental genes sFab (codon-optimized) and Fab0 (harmonized). The five altered segments (the numbers on top) are indicated by red tiles and base-pair numbers (counted from the start codon in the light chain signal sequence), and include: 1. The start of variable light region (de-optimization of codon pair), 2. Frame2-CDRL2-frame3 of variable light region (harmonization), 3. Constant light region (harmonization), 4. PelB signal sequence (de-optimization of leucine codon pair) and 5. Constant heavy region (harmonization). (**b**) Chimeric variants obtained by replacing one of the codon harmonized segments in Fab0 with the corresponding codon-optimized sequence (indicated by white tiles with black outline).

**Figure 2:**
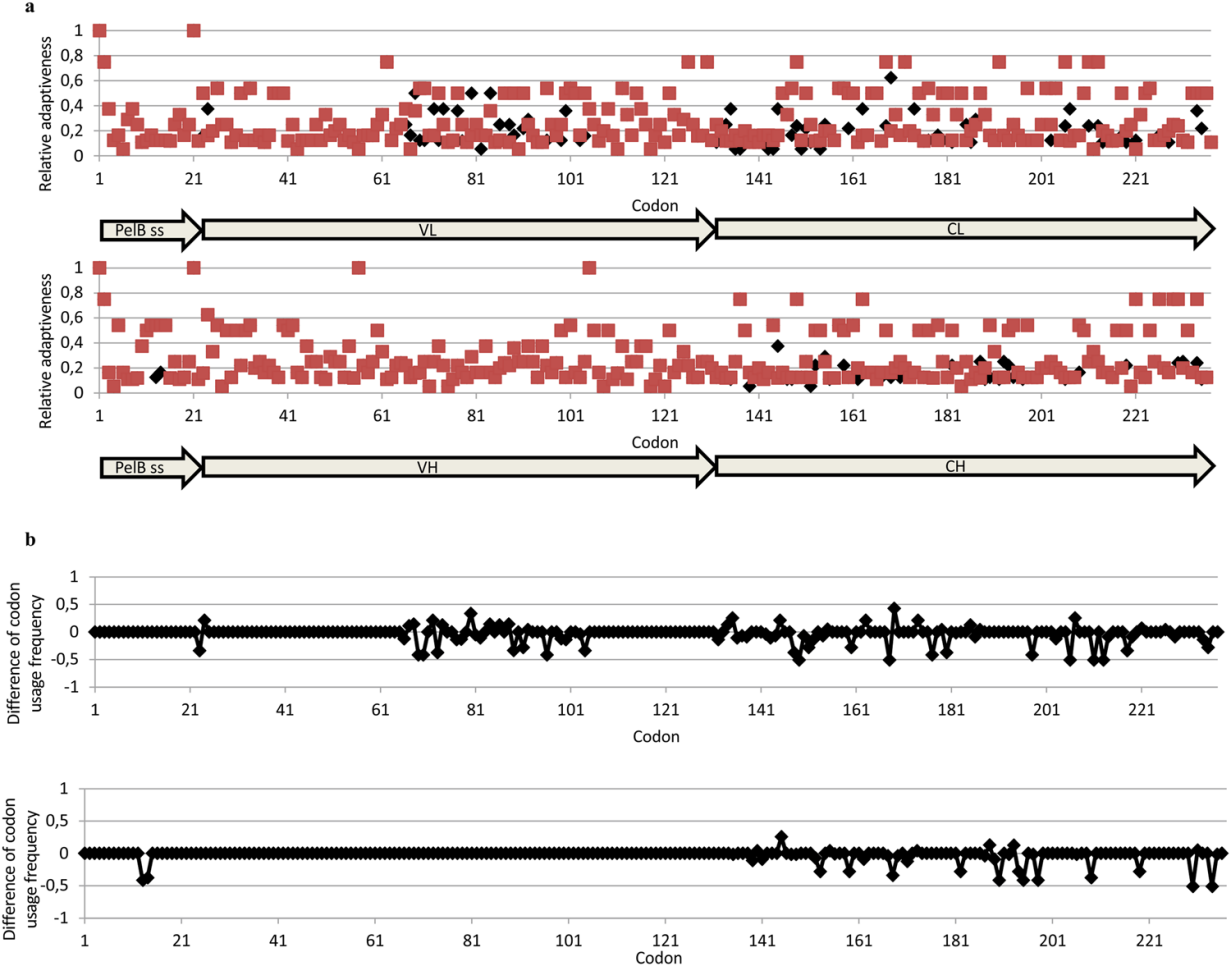
Codon usage of sFab and Fab0. In both figures, the light chain is represented in the upper and the heavy chain in the lower diagram. (**a**) The relative adaptiveness diagrams showing the codon usage of sFab (red) and Fab0 (black). (**b**) Diagrams showing the difference of codon usage frequency between sFab and Fab0. Peaks above zero indicate that a codon of Fab0 has higher frequency than the corresponding sFab codon and *vice versa*.

**Figure 3:**
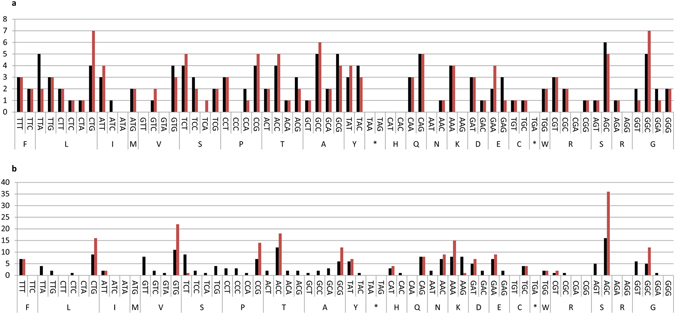
The total codon counts in the variable segments and the constant segments of Fab0 and sFab. (**a**) Codon counts in the variable segments and (**b**) the constant segments. The black bars show the count of each codon in Fab0 and red bars in sFab.

The anti-digoxigenin antibody fragment used in this study was originally isolated from a synthetic ScFv phage display library^30^ and subsequently converted into a Fab format. The inserted human light (C_K_) and heavy chain (C_H1_) constant domains were optimized by the “one amino acid-one codon” method, in which the most frequent codon of *E. coli* is used to encode every amino acid. Analysis of the codon usage of the constant domains was performed by using tAI. The resulted tAI-values were 0.24 and 0.23 for C_K_ and C_H1_. Only 18 codons of 61 were used to code for the optimized C_H1_ (does not contain methionine nor arginine residues) and 20 codons of 61 for the optimized C_K_ (does not contain methionine). In functional testing, the resulting sFab construct proved to be extremely lethal to *E. coli* cells (Table 1). In order to restore cell viability and Fab production, several DNA segments of the Fab gene were then redesigned with synonymous codon substitutions (Supplementary Fig. 2a). The C_K_, C_H1_ and a part of the variable light domain (V_L_), amino acid residues 66–104 (as counted from the first residue of the preceding PelB signal sequence, or residues 49–97 by IMGT numbering), were harmonized by the method presented by Angov *et al*.^16^. In the Angov method the heterologous gene is engineered with synonymous codons to obtain a similar positional codon usage profile as is present in the original host. Consequently, in the Angov method, other codons apart from the most frequent are also used, unlike in the “one amino acid-one codon” method. Implementation of the Angov method resulted in the diversification of the codon usage in constant domains leading to the use of 40/61 codons for coding the C_H1_ and 44/61 for coding the C_K_. The parental sFab C_K_ and C_H1_ domains will be referred to as “over-optimized” in contrast to the harmonized versions. In addition, codons were de-optimized at the beginning of the V_L_ domain and in the PelB signal sequence of the heavy chain. These changes were inspired by the observation that the first codons of a reading frame are often translated with lower efficiency than other parts of the gene^40^. The resulting Fab construct was termed Fab0 (containing all changes). All created constructs are illustrated in Fig. 1a. The variants 1, 2, 3, 4 and 5 were produced by changing the sFab segments one-by-one back to the Fab0 in order to study the effect of individual segments (Fig. 1b and Supplementary Fig. 2b). The relative adaptiveness of codons of sFab and Fab0 genes is shown in Fig. 2a and the difference between sFab and Fab0 codon usage frequency in Fig. 2b. The difference of codon usage frequency was calculated by subtracting the relative adaptiveness value of the codon in the sFab gene from the value of the corresponding codon in the Fab0 gene. The tAI-values for the parent genes Fab0 and sFab were 0.21 and 0.23 and for the variants 1, 2, 3, 4 and 5 these values were 0.21, 0.21, 0.22, 0.21 and 0.21, respectively. The local tAI-values for the codon-optimized segments 1, 2, 3, 4 and 5 were 0.29, 0.24, 0.24, 0.54 and 0.23. Corresponding local tAI-values for the codon harmonized segments were 0.24, 0.20, 0.20, 0.14 and 0.20. Hence, the codon harmonizations lead to the decrease in local tAI-values for every segment. Moreover, the codon harmonization of C_K_ and C_H1_ lead to more diverse usage of codons in these domains. The count of each codon in the variable and constant segments of Fab0 and sFab are shown in the Fig. 3a and b.

**Table 1:** The average generation times.

The average generation times (min)					
Variant/Parent	XL1-Blue			BL21	
	pAK400	pEB32x	pLK04	pAK400	pLK04
Fab0	45 (±0.8)	37 (±1.2)	56 (±2.2)	53 (±4.2)	109 (±11.3)
sFab	No growth	No growth	N.D.	N.D.	N.D.
Variant 1	45 (±2.2)	40 (±4.5)	105 (±73.6)^a^	51 (±5.7)	107 (±18.4)
Variant 2	98 (±6.2)^a^	42 (±5.0)	80 (±31.4)	67 (±2.1)	106 (±16.3)
Variant 3	77 (±17.1)^a^	90 (±44.3)^a^	No growth	44 (±6.4)	No growth
Variant 4	45 (±4.5)	37 (±1.2)	88 (±21.1)^a^	64 (±11.3)	66 (±17.7)
Variant 5	47 (±0.8)	44 (±10.7)	85 (±39.0)	56 (±2.1)	108 (±19.1)

The average generation times of the parent genes and the variants from three independent cultures are shown in the table. The standard deviations of the generation times are shown in parentheses. The variants that differ statistically significantly (Mann-Whitney U test) from Fab0 are marked by the letter “a”. “No growth” means that the parent or the variant could not be cloned or cultivated in liquid culture. N.D. = not determined.

The mRNA minimum free energy (ΔG) for the parent genes sFab and Fab0 and for the variants 1, 2, 3, 4 and 5 were −594.7, −539.0, −536.1, −548.9, −552.9, −546.1 and −562.2 kcal/mole. Respective GC percentages were 56.85%, 53.57%, 53.34%, 53.93%, 54.65%, 53.79% and 55.34%. The codon harmonization increased the mRNA minimum free energy (ΔG), thus reducing the stability of the mRNA secondary structures. The GC percentage was also decreased. In most cases, excluding variant 1, addition of the codon-optimized segment into the codon harmonized parent gene Fab0 decreased both parameters indicating that the more stable secondary structure of sFab mRNA was a cumulative effect of several over-optimized segments.

### Effect of codon usage on cell fitness

Effect of codon usage alteration on cell fitness was studied by cultivating XL1-Blue and BL21 cells harboring different vector, variant and parent gene combinations and observing the growth of cells. The Fab genes were introduced in the cells in two similar vectors intended for the expression of soluble Fab. The vectors differed only by the antibiotic resistance marker, which was chloramphenicol acetyltransferase in pAK400 and ß-lactamase in pLK04. Furthermore, fitness was measured with cells carrying phagemid pEB32x expressing Fab variants as a fusion to the C-terminal part of a g3pΔ. The average generation times were calculated from three independent cultures. The effect of codon usage was evaluated by comparing the generation times of the variants to the generation time of the parent gene Fab0. The variant 3 exhibited exclusively negative fitness effects in XL1-Blue strain (n = 3, 2-tailed *p* = 0.05) in both the chloramphenicol-resistant (CAT) phage display vector pEB32x and the soluble expression vector pAK400 (CAT) (n = 3, 2-tailed *p* = 0.05) (Table 1). Cloning of the variant 3 into the ampicillin resistant pLK04 vector failed despite six independent attempts, indicating that over-optimized C_K_ has a strong detrimental effect on cell growth. Interestingly, the variant 5 containing the over-optimized C_H1_ did not have a similar retarding effect on cell growth as the variant 3, although the difference in tAI-values between over-optimized and harmonized constant domains was similar in both cases. Variant 2 showed significant negative fitness effects (n = 3, 2-tailed *p* = 0.05) only in pAK400/XL-1 and the variants 1 (n = 3, 2-tailed *p* = 0.05) and 4 (n = 3, 2-tailed *p* = 0.05) only as pLK04/XL-1 combination, respectively (Table 1). None of the chimeric variants 1–5 showed significantly positive fitness effects. There was overall less fitness differences between the Fab variants in the *E. coli* B strain BL21 (Table 1). This is partly explained by the longer generation time of the parental Fab0 clone in BL21 than in XL-1 Blue (8 min longer with pAK400 and 53 min with pLK04). In general, the cells transformed with the display vector expressing Fab-g3pΔ fusion protein were the least negatively affected.

### Effect of codon usage alteration on the total yield of immunoreactive Fab fragment

The influence of codon usage alteration on the total yield of immunoreactive Fab fragment was studied by expressing the variants and both parent genes from the soluble expression vectors pAK400 and pLK04 in *E. coli* strains XL1-Blue and BL21. The yields were measured after 3 h of induction by an immunoassay using solid-phase bound biotinylated digoxigenin to capture the Fab and labeled anti-human Fab antibody as a tracer. The average yields of immunoreactive Fab fragment from three independent expressions are presented in Table 2. The total Fab yield, the sum of intracellular and extracellular Fab, obtained from the variants was compared to the total yield obtained from the parental gene Fab0 in pairwise fashion. The sampling size in the case of each parent/variant results from three independent expressions from which three parallel samples were taken to the immunoassay analysis (3 × 3 = 9). In general, protein yields obtained from BL21 strain were higher compared to XL1-Blue. According to this analysis the most detrimental change for protein production was the codon optimization of the constant light region as also observed in the cell growth analysis. In pAK400 vector the total expression levels of the variant 3 decreased 82% in XL1-Blue strain (n = 9, 2-tailed *p* = 0.0002) and 82% in BL21 strain (n = 6, 2-tailed *p* = 0.001) as compared to Fab0. The effect of the chosen resistance gene marker was most strikingly observed by expressing the variants 2 and 5 in XL1-Blue host. In the chloramphenicol-resistant pAK400 vector, the variant 2 that contained synonymous codon alterations in the V_L_ domain, yielded 79% (n = 9, 2-tailed *p* = 0.0002) less protein than the parental Fab0, whereas in the ampicillin resistant pLK04 vector the variant 2 yielded 111% (n = 9, 2-tailed *p* = 0.0002) higher expression than the parental Fab0. The situation changed when BL21 was used as a host instead of XL1-Blue (Table 2). With pLK04/BL21 combination, the expression levels of the variant 2 were significantly (n = 9, 2-tailed *p* = 0.005) lower than the parental gene Fab0 and slightly higher with pAK400/BL21. The total expression of the variant 5 in XL1-Blue using pAK400 was similar to Fab0, but in pLK04 vector the expression decreased to a level that could not be reliably detected. Two of the variants, 1 and 4, exhibited mostly insignificant effects on the total yield. However, both showed significantly positive effects with one combination. The variant 4 exhibited significantly (n = 9, 2-tailed *p* = 0.002) positive effects with the combination of pLK04 vector and XL1-Blue strain, whereas the variant 1 exhibited significantly (n = 9, 2-tailed *p* = 0.04) positive effects with the combination of pAK400 vector and BL21 strain (Table 2).

**Table 2:** The average yields of immunoreactive Fab fragment.

The average yields of immunoreactive Fab fragment (µg/ml)												
Variant/Parent	XL1-Blue						BL21					
	pAK400			pLK04			pAK400			pLK04		
	Cells	Supernatant	Total	Cells	Supernatant	Total	Cells	Supernatant	Total	Cells	Supernatant	Total
Fab0	2.59 (±1.04)	0.03 (±0.01)	2.62 (±1.04)	1.64 (±0.21)	0.04 (±0.005)	1.68 (±0.21)	5.97 (±0.86)	0.18 (±0.05)	6.15 (±0.90)	7.46 (±2.71)	0.34 (±0.11)	7.80 (±2.78)
sFab	N.D.	N.D.	N.D.	N.D.	N.D.	N.D.	N.D.	N.D.	N.D.	N.D.	N.D.	N.D.
Variant 1	2.79 (±0.82)	0.04 (±0.01)	2.83 (±0.83)	1.83 (±0.86)	0.12 (±0.08)	1.94 (±0.84)	7.42 (±1.58)	0.30 (±0.07)	7.72 (±1.61)	7.52 (±2.20)	0.40 (±0.06)	7.92 (±2.26)
Variant 2	0.35 (±0.07)	0.23 (±0.06)	0.58 (±0.12)	3.49 (±1.32)	0.05 (±0.02)	3.54 (±1.32)	7.36 (±1.87)	0.33 (±0.11)	7.69 (±1.96)	2.36 (±3.21)	0.13 (±0.15)	2.49 (±3.38)
Variant 3	0.46 (±0.04)	0.01 (±0.001)	0.47 (±0.04)	N.D.	N.D.	N.D.	1.08 (±0.26)	<0.0015	1.08 (±0.26)	N.D.	N.D.	N.D.
Variant 4	2.87 (±1.55)	0.03 (±0.01)	2.9 (±1.55)	2.26 (±0.43)	0.14 (±0.06)	2.40 (±0.39)	7.02 (±1.49)	0.24 (±0.11)	7.26 (±1.48)	8.67 (±3.98)	0.33 (±0.16)	9.00 (±4.09)
Variant 5	2.14 (±0.53)	0.04 (±0.01)	2.18 (±0.53)	<0.0015	<0.0015	<0.0015	6.61 (±1.29)	0.55 (±0.03)	7.16 (±1.27)	6.84 (±3.04)	0.40 (±0.23)	7.24 (±3.23)

The average yields of immunoreactive Fab fragment of the parent genes and the variants from three independent cultures are shown in the table. The standard deviations of the yields are shown in parentheses. N.D. = not determined.

### Effect of mRNA minimum free energy (ΔG) on expression levels

The connection between the mRNA minimum free energy (ΔG) and the expression levels was analyzed by the Spearman’s rank correlation. The correlations were determined separately for each of the four vector/strain combination. Each vector/strain combination included the parent gene Fab0 and the variants, which differed significantly from the parent gene Fab0 by their yields of immunoreactive Fab with a given combination. Thus, the variants 2 and 3 (pAK400/XL1-Blue), the variants 2 and 4 (pLK04/XL1-Blue), the variants 1 and 3 (pAK400/BL21) and the variant 2 (pLK04/BL21) were included in the case of each combination, in addition to the parent Fab0. The parent and each variant has an n value of 9 (excluding the variant 3 in pAK400/BL21), for the reason explained above. Therefore, for example in the case of pAK400/XL1-Blue, the number of total data points is 27.

**Figure 4:**
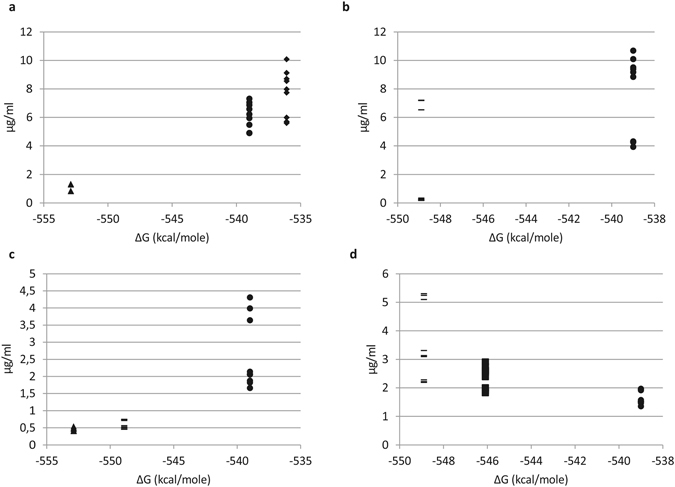
Correlation between mRNA minimum free energy (ΔG) and protein yield. (**a**) BL21/pAK400 combination. (**b**) BL21/pLK04 combination. (**c**) XL1-Blue/pAK400 combination. (**d**) XL1-Blue/pLK04 combination. Data points are marked as follows: the parent Fab0 (sphere) and the variants 1 (diamond), 2 (line), 3 (triangle) and 4 (square).

Statistically significant correlations were observed with all combinations. In most cases, the results suggest that when the predicted mRNA minimum free energy (ΔG) decreases, the expression levels also decrease. This is in good agreement with earlier literature showing that high protein expression favors weaker structural stability of the mRNA, for example near the translation start site^20, 22^. The strong positive correlation between ΔG and protein expression was observed in BL21 with both pAK400 (n = 24, r = 0.664, 2-tailed *p* = 0.0002) and pLK04 (n = 18, r = 0.675, 2-tailed *p* = 0.002) and in XL1-Blue with pAK400 (n = 27, r = 0.827, 2-tailed *p* = 0.0002). In contrast, pLK04/XL1-Blue combination expressed very strong negative correlation (n = 27, r = −0.804, 2-tailed *p* = 0.0002). In this case, decrease in mRNA minimum free energy (ΔG) seemed to increase the expression levels. Correlations between mRNA minimum free energy (ΔG) and protein yield are illustrated also as scatter plots in Fig. 4a–d.

### Up-scaled protein production

The harmonized parental gene Fab0 was expressed from pLK04 vector in BL21 strain in shake flask culture (2 × 200 ml). After 4 h of induction, when the OD (600 nm) of the cultures were 3.7 and 4.0, cells were isolated from 1 ml samples for the assessment of the Fab levels. The concentrations of functional Fab, analyzed by the immunoassay as described above, were 11.0 and 9.5 mg/L in the two cultures giving a total Fab yield of 4 mg.

### Effect of codon usage alteration on phage immunoreactivity

**Figure 5:**
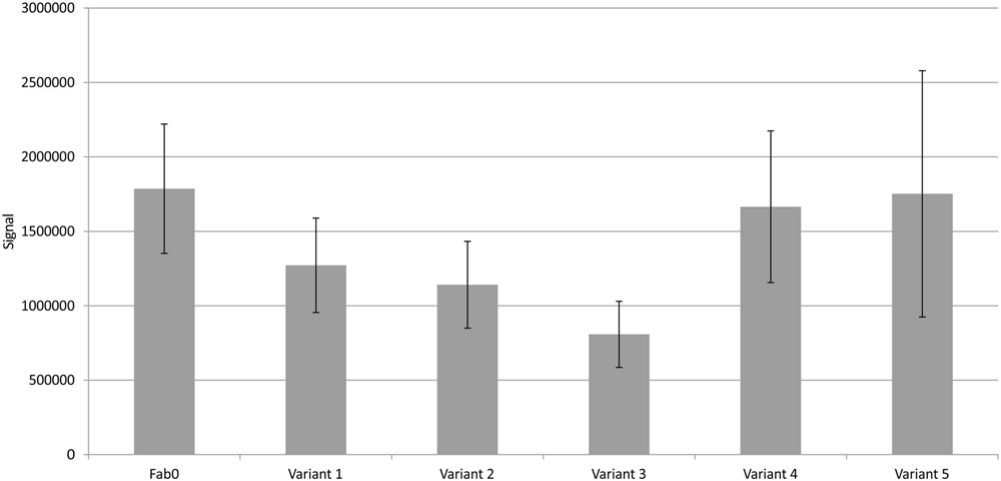
Immunoreactivity of Fab displaying phage towards immobilized digoxigenin. The average signals of the parent gene Fab0 and the variants 1–5 from three phage productions are represented.

Influence of codon usage alteration on filamentous phage display efficiency was studied by displaying all variants and both parent genes as a fusion to g3pΔ using phagemid vector pEB32x, and measuring the immunoreactivity of the Fab phage. Phage productions were repeated three times. The number of phage particles (phage titer) in each production was determined by OCCA method and according to the results, normalized to 1 × 10^9^ pfu per sample. The binding of phage to the immobilized digoxigenin was analyzed by phage immunoassay, the results of which are shown in the Fig. 5. Statistically significant decreases in phage immunoreactivity were observed in the cases of variant 2 (2-tailed *p* = 0.012) and variant 3 (2-tailed *p* = 0.002) when compared to the parent gene Fab0 (Fig. 5). No statistically significant increases were observed.

## Discussion

In the current study, the codon-optimized synthetic human Fab gene (sFab) was redesigned by making synonymous codon substitutions to five segments of the Fab gene framework. There was a striking difference between the phenotype of the harmonized Fab0 and codon-optimized sFab, since the latter could not be cultivated on agar plate or in liquid culture. Harmonized Fab gene framework (Fab0) restored cell viability, recombinant protein production and phage display capability. The positive effect of codon harmonization on heterologous protein production has been described earlier for proteins expressed in the cytoplasm of *E. coli*. The mechanism for the improved expression has been suggested to be related to protein folding by mimicking the translation kinetics in the native host^16, 41, 42^. In this study we show that harmonization method is successful also with a secreted protein. We were able to increase the expression of the Fab fragment from negligible levels to 10 mg/l.

The exploration of the contribution of the individual harmonized DNA segments on the Fab expression revealed that the codon-optimization of the light chain segments had the most deleterious effects, which is in-line with previous observation made by Lin *et al*.^12^. The effects of light chain alterations were not only confined to the yield of soluble Fab, but extended also to phage immunoreactivity (Fig. 5). Among the altered light chain segments, the C_K_ domain segment was especially susceptible to codon-optimization (variant 3). The other constant domain segment, C_H1_, did not show equally deleterious effects on protein yield and cell fitness upon the change in the codon usage (variant 5). The variant 5 had lower mRNA minimum free energy (ΔG) than the variant 3, although generally, lower mRNA minimum free energy (ΔG) (increasing stability of the secondary structure of mRNA) correlated with decreasing expression levels. In addition to expression differences, both codon-optimized constant domain segments exhibited very different effects on cell fitness. In contrast to variant 5, the negative effect of the variant 3 on protein yield was accompanied by reduced fitness of the cells with all vector-host-combinations. In general, improved fitness of the cells predicted higher protein expression levels. The exceptions were variants 1, 4 and 5 with the pLK04/XL1-Blue combination and the variant 3 with the pAK400/BL21 combination.

The cloning of a panel of synonymously coded Fab fragments into the vectors pAK400 and pLK04 offered an intriguing opportunity to investigate the interplay between codon usage and antibiotic resistance as the only difference between the vectors was the antibiotic resistance marker. The change of vector from pAK400 to pLK04 caused a striking decrease in the expression levels of the variants 2 and 5, and, in the case of variant 3, viable transformants were only obtained with pAK400. With variants 2 and 5, the effect was strain dependent and observed in the K-12 type strain XL1-Blue, a common cloning and phage display host, but not in the B-type BL21, intended for protein expression. The strains differ from each other in many respects^43^, and the specific reason behind the observed effects remains to be resolved. For example, B strain has an additional type II secretion system and different cell wall and outer membrane composition, whereas K-12 shows higher expression of heat shock genes^43^ and XL1-Blue specifically carries mutations helping to maintain plasmid integrity, as well as to avoid stringent response during amino acid deprivation^44^.

In conclusion, our results show the expression of a hetero dimeric secretory protein can be improved in *E. coli* from negligible levels to 10 mg/l by harmonizing selected DNA segments by altering synonymous codons usage. Furthermore, the study shows that the effects of codon bias are not easily predictable, but may lead to different outcomes depending on the host strain or the chosen antibiotic resistance marker. Our observations reveal the additional complexity involved in heterologous protein expression suggesting that one gene design does not necessarily produce the best outcome in all conditions.

## Electronic supplementary material

Supplementary information
