# Supplementary material for: Effect of DNA sequence of Fab fragment on yield characteristics and cell growth of E. coli
Source: Sci Rep. 2017 Jun 19;7:3796. doi: 10.1038/s41598-017-03957-6 (PMC5476587; doi:10.1038/s41598-017-03957-6)
Supplement: Supplementary file 1 — Supplementary information [file 41598_2017_3957_MOESM1_ESM.pdf]

Effect of DNA sequence of Fab fragment on yield characteristics and cell growth of *E. coli*

Antti Kulmala[a], Tuomas Huovinen[b] and Urpo Lamminmäki[a]

[a]Department of Biochemistry/Biotechnology, University of Turku, 20520 Turku, Finland

[b]Department of Biochemistry, University of Cambridge, Cambridge CB2 1GA, UK

Supplementary figures

1 2
MKYLLPTAAAGLLLLAAQPAMA¦EIVLTQSPGTLSLSPGERATLSCRASQSVSSSSLDWYQQKPGQ

APRLLIYGASSRATGVPDRFSGSGSGTDFTLTISRLEPEDFAVYYCLQWNYFPYTFGQGTKVEIKR

3
¦TVAAPSVFIFPPSDEQLKSGTASVVCLLNNFYPREAKVQWKVDNALQSGNSQESVTEQDSKDSTY

4
SLSSTLTLSKADYEKHKVYACEVTHQGLSSPVTKSFNRGES¦*SRLIKGELN¦MKYLLPTAAAGLL

5
LLAAAPAMA¦EVQLLESGGGLVQPGGSLRLSCAASGFTFSSYAMNWVRQAPGKGLEWVSQINPSGG

STYYADSVKGRFTISRDNSKNTLYLQMNSLRAEDTAVYYCVGHEWGQGTLVTVSSA¦STKGPSVFP

6
LAPSSKSTSGGTAALGCLVKDYFPEPVTVSWNSGALTSGVHTFPAVLQSSGLYSLSSVVTVPSSSL

GTQTYICNVNHKPSNTKVDKKVEPKSS¦

Supplementary figure S1. The amino acid sequence of the Fab fragments. Both parent genes and all variants produce the same amino acid sequence. Different segments of the Fab fragment are separated by the vertical broken lines. Segments in order are: 1. The PelB signal sequence of the light chain, 2. The variable region of the light chain, 3. The constant region of the light chain, 4. The PelB signal sequence of the heavy chain, 5. The variable region of the heavy chain, 6. The constant region of the heavy chain.

a

sFab

1
ATGAAATACCTATTGCCTACGGCAGCCGCTGGATTGTTATTACTCGCGGCCCAGCCGGCCATGGCG¦GAAATTGTGCTGA

2
CCCAATCTCCGGGCACACTGAGCTTGTCTCCGGGCGAACGTGCGACCCTTAGCTGCAGAGCCAGCCAGTCGGTGTCCAGC

TCGTCTCTTGATTGGTATCAACAGAAACCAGGTCAAGCACCTCGCCTGCTGATTTATGGCGCCTCTTCACGTGCCACTGG

GGTCCCGGATCGCTTTAGCGGCTCTGGCAGTGGCACCGATTTTACTCTGACCATTTCCCGTCTGGAACCGGAAGACTTCG

CGGTGTACTATTGTCTGCAGTGGAACTACTTCCCTTATACCTTTGGCCAGGGGACGAAAGTCGAGATTAAACGG¦ACCGT

3
GGCGGCGCCGAGCGTGTTTATTTTTCCGCCGAGCGATGAACAGCTGAAAAGCGGCACCGCGAGCGTGGTGTGCCTGCTGA

ACAACTTTTATCCGCGTGAAGCGAAAGTGCAGTGGAAAGTGGATAACGCGCTGCAGAGCGGCAACAGCCAGGAAAGCGTG

ACCGAACAGGATAGCAAAGATAGCACCTATAGCCTGAGCAGCACCCTGACCCTGAGCAAAGCGGATTATGAAAAACATAA

AGTGTATGCGTGCGAAGTGACCCATCAGGGCCTGAGCAGCCCGGTGACCAAGAGCTTTAACCGTGGCGAATCT¦TAATCT

4

AGATTAATTAAAGGAGAATTGAAT¦ATGAAATATCTTCTGCCGACTGCTGCGGCAGGCCTGCTGCTGCTGGCGGCCGCTC

CAGCCATGGCT¦GAGGTACAGCTGCTTGAAAGCGGCGGTGGCCTGGTGCAACCGGGTGGGAGCCTGCGTCTGTCGTGCGC

AGCCTCCGGATTTACGTTCTCCAGCTACGCGATGAATTGGGTCCGTCAGGCTCCAGGTAAGGGTCTCGAGTGGGTCAGTC

AAATCAATCCTTCTGGTGGGAGCACGTATTATGCAGATAGCGTGAAGGGTCGCTTCACCATCTCCCGGGACAATTCGAAG

AACACACTGTATCTCCAAATGAACTCGCTTCGTGCTGAGGACACTGCCGTCTACTACTGTGTTGGTCATGAATGGGGTCA

6

GGGTACACTAGTCACCGTGAGCTCGGCG¦AGCACCAAAGGCCCGAGCGTGTTTCCGCTGGCGCCGAGCAGCAAAAGCACC

AGCGGCGGCACCGCGGCGCTGGGCTGCCTGGTGAAAGATTATTTTCCGGAACCGGTGACCGTGAGCTGGAACAGCGGCGC

GCTGACCAGCGGCGTGCATACCTTTCCGGCGGTGCTGCAGAGCAGCGGCCTGTATAGCCTGAGCAGCGTGGTGACCGTGC

CGAGCAGCAGCCTGGGCACCCAGACCTATATTTGCAACGTGAACCATAAACCGAGCAACACCAAAGTGGATAAAAAAGTG

GAACCGAAAAGCAGC ¦

Fab0

1

ATGAAATACCTATTGCCTACGGCAGCCGCTGGATTGTTATTACTCGCGGCCCAGCCGGCCATGGCG¦GAGATCGTGCTGA

2

CCCAATCTCCGGGCACACTGAGCTTGTCTCCGGGCGAACGTGCGACCCTTAGCTGCAGAGCCAGCCAGTCGGTGTCCAGC

TCGTCTCTTGATTGGTATCAACAGAAACCAGGTCAAGCCCCGCGTTTATTAATTTACGGAGCATCTAGCCGCGCGACTGG

CGTGCCTGATCGTTTTTCCGGCTCCGGGAGTGGTACGGATTTTACTTTAACCATTAGCCGCCTGGAACCAGAGGACTTCG

CGGTGTACTATTGTCTGCAGTGGAACTACTTCCCTTATACCTTTGGCCAGGGGACGAAAGTCGAGATTAAACGG¦ACTGT

3

GGCCGCACCTAGTGTTTTTATTTTTCCCCCTAGTGACGAACAGTTGAAGAGTGGTACTGCGAGTGTCGTGTGCCTGCTGA

ATAACTTTTACCCGCGTGAAGCGAAGGTACAGTGGAAAGTGGACAACGCGTTACAGTCGGGAAACTCTCAGGAATCCGTT

ACGGAACAGGATAGCAAAGATAGCACCTATAGCTTAAGCAGCACCCTGACACTGAGCAAGGCAGATTATGAAAAGCATAA

GGTTTATGCGTGCGAGGTTACCCACCAGGGCCTGAGCTCGCCGGTTACCAAGAGCTTTAACCGCGGTGAATCTTAG¦TCT

4

AGATTAATTAAAGGAGAATTGAAT¦ATGAAATATCTTCTGCCGACTGCTGCGGCAGGCCTGTTATTGCTGGCGGCCGCTC

5

CAGCCATGGCT¦GAGGTACAGCTGCTTGAAAGCGGCGGTGGCCTGGTGCAACCGGGTGGGAGCCTGCGTCTGTCGTGCGC

AGCCTCCGGATTTACGTTCTCCAGCTACGCGATGAATTGGGTCCGTCAGGCTCCAGGTAAGGGTCTCGAGTGGGTCAGTC

AAATCAATCCTTCTGGTGGGAGCACGTATTATGCAGATAGCGTGAAGGGTCGCTTCACCATCTCCCGGGACAATTCGAAG

AACACACTGTATCTCCAAATGAACTCGCTTCGTGCTGAGGACACTGCCGTCTACTACTGTGTTGGTCATGAATGGGGTCA

6

GGGTACACTAGTCACCGTGAGCTCGGCG¦TCTACCAAAGGCCCTTCGGTTTTTCCGCTGGCACCGTCTTCTAAAAGCACC

AGTGGTGGCACGGCTGCGCTGGGTTGCCTGGTTAAAGATTATTTTCCAGAGCCCGTGACAGTGTCGTGGAACTCAGGCGC

GCTGACCAGCGGTGTGCATACCTTTCCCGCCGTTCTCCAGTCTTCCGGTTTATATTCTTTAAGCAGCGTGGTGACCGTGC

CGTCTAGCAGCTTGGGCACCCAGACCTATATTTGCAACGTGAATCATAAACCGAGCAACACCAAAGTGGATAAAAAGGTC
GAACCGAAGTCTAGC¦

b

Variant 1

ATGAAATACCTATTGCCTACGGCAGCCGCTGGATTGTTATTACTCGCGGCCCAGCCGGCCATGGCGGAAATTGTGCTGAC
CCAATCTCCGGGCACACTGAGCTTGTCTCCGGGCGAACGTGCGACCCTTAGCTGCAGAGCCAGCCAGTCGGTGTCCAGCT
CGTCTCTTGATTGGTATCAACAGAAACCAGGTCAAGCCCCGCGTTTATTAATTTACGGAGCATCTAGCCGCGCGACTGGC
GTGCCTGATCGTTTTTCCGGCTCCGGGAGTGGTACGGATTTTACTTTAACCATTAGCCGCCTGGAACCAGAGGACTTCGC
GGTGTACTATTGTCTGCAGTGGAACTACTTCCCTTATACCTTTGGCCAGGGGACGAAAGTCGAGATTAAACGGACTGTGG
CCGCACCTAGTGTTTTTATTTTTCCCCCTAGTGACGAACAGTTGAAGAGTGGTACTGCGAGTGTCGTGTGCCTGCTGAAT
AACTTTTACCCGCGTGAAGCGAAGGTACAGTGGAAAGTGGACAACGCGTTACAGTCGGGAAACTCTCAGGAATCCGTTAC
GGAACAGGATAGCAAAGATAGCACCTATAGCTTAAGCAGCACCCTGACACTGAGCAAGGCAGATTATGAAAAGCATAAGG
TTTATGCGTGCGAGGTTACCCACCAGGGCCTGAGCTCGCCGGTTACCAAGAGCTTTAACCGCGGTGAATCTTAGTCTAGA
TTAATTAAAGGAGAATTGAATATGAAATATCTTCTGCCGACTGCTGCGGCAGGCCTGTTATTGCTGGCGGCCGCTCCAGC
CATGGCTGAGGTACAGCTGCTTGAAAGCGGCGGTGGCCTGGTGCAACCGGGTGGGAGCCTGCGTCTGTCGTGCGCAGCCT
CCGGATTTACGTTCTCCAGCTACGCGATGAATTGGGTCCGTCAGGCTCCAGGTAAGGGTCTCGAGTGGGTCAGTCAAATC
AATCCTTCTGGTGGGAGCACGTATTATGCAGATAGCGTGAAGGGTCGCTTCACCATCTCCCGGGACAATTCGAAGAACAC
ACTGTATCTCCAAATGAACTCGCTTCGTGCTGAGGACACTGCCGTCTACTACTGTGTTGGTCATGAATGGGGTCAGGGTA
CACTAGTCACCGTGAGCTCGGCGTCTACCAAAGGCCCTTCGGTTTTTCCGCTGGCACCGTCTTCTAAAAGCACCAGTGGT
GGCACGGCTGCGCTGGGTTGCCTGGTTAAAGATTATTTTCCAGAGCCCGTGACAGTGTCGTGGAACTCAGGCGCGCTGAC
CAGCGGTGTGCATACCTTTCCCGCCGTTCTCCAGTCTTCCGGTTTATATTCTTTAAGCAGCGTGGTGACCGTGCCGTCTA
GCAGCTTGGGCACCCAGACCTATATTTGCAACGTGAATCATAAACCGAGCAACACCAAAGTGGATAAAAAGGTCGAACCG
AAGTCTAGC

Variant 2

ATGAAATACCTATTGCCTACGGCAGCCGCTGGATTGTTATTACTCGCGGCCCAGCCGGCCATGGCGGAGATCGTGCTGAC
CCAATCTCCGGGCACACTGAGCTTGTCTCCGGGCGAACGTGCGACCCTTAGCTGCAGAGCCAGCCAGTCGGTGTCCAGCT
CGTCTCTTGATTGGTATCAACAGAAACCAGGTCAAGCACCTCGCCTGCTGATTTATGGCGCCTCTTCACGTGCCACTGGG
GTCCCGGATCGCTTTAGCGGCTCTGGCAGTGGCACCGATTTTACTCTGACCATTTCCCGTCTGGAACCGGAAGACTTCGC
GGTGTACTATTGTCTGCAGTGGAACTACTTCCCTTATACCTTTGGCCAGGGGACGAAAGTCGAGATTAAACGGACTGTGG
CCGCACCTAGTGTTTTTATTTTTCCCCCTAGTGACGAACAGTTGAAGAGTGGTACTGCGAGTGTCGTGTGCCTGCTGAAT
AACTTTTACCCGCGTGAAGCGAAGGTACAGTGGAAAGTGGACAACGCGTTACAGTCGGGAAACTCTCAGGAATCCGTTAC
GGAACAGGATAGCAAAGATAGCACCTATAGCTTAAGCAGCACCCTGACACTGAGCAAGGCAGATTATGAAAAGCATAAGG
TTTATGCGTGCGAGGTTACCCACCAGGGCCTGAGCTCGCCGGTTACCAAGAGCTTTAACCGCGGTGAATCTTAGTCTAGA
TTAATTAAAGGAGAATTGAATATGAAATATCTTCTGCCGACTGCTGCGGCAGGCCTGTTATTGCTGGCGGCCGCTCCAGC
CATGGCTGAGGTACAGCTGCTTGAAAGCGGCGGTGGCCTGGTGCAACCGGGTGGGAGCCTGCGTCTGTCGTGCGCAGCCT
CCGGATTTACGTTCTCCAGCTACGCGATGAATTGGGTCCGTCAGGCTCCAGGTAAGGGTCTCGAGTGGGTCAGTCAAATC
AATCCTTCTGGTGGGAGCACGTATTATGCAGATAGCGTGAAGGGTCGCTTCACCATCTCCCGGGACAATTCGAAGAACAC
ACTGTATCTCCAAATGAACTCGCTTCGTGCTGAGGACACTGCCGTCTACTACTGTGTTGGTCATGAATGGGGTCAGGGTA
CACTAGTCACCGTGAGCTCGGCGTCTACCAAAGGCCCTTCGGTTTTTCCGCTGGCACCGTCTTCTAAAAGCACCAGTGGT
GGCACGGCTGCGCTGGGTTGCCTGGTTAAAGATTATTTTCCAGAGCCCGTGACAGTGTCGTGGAACTCAGGCGCGCTGAC
CAGCGGTGTGCATACCTTTCCCGCCGTTCTCCAGTCTTCCGGTTTATATTCTTTAAGCAGCGTGGTGACCGTGCCGTCTA
GCAGCTTGGGCACCCAGACCTATATTTGCAACGTGAATCATAAACCGAGCAACACCAAAGTGGATAAAAAGGTCGAACCG
AAGTCTAGC

Variant 3

ATGAAATACCTATTGCCTACGGCAGCCGCTGGATTGTTATTACTCGCGGCCCAGCCGGCCATGGCGGAGATCGTGCTGAC
CCAATCTCCGGGCACACTGAGCTTGTCTCCGGGCGAACGTGCGACCCTTAGCTGCAGAGCCAGCCAGTCGGTGTCCAGCT
CGTCTCTTGATTGGTATCAACAGAAACCAGGTCAAGCCCCGCGTTTATTAATTTACGGAGCATCTAGCCGCGCGACTGGC
GTGCCTGATCGTTTTTCCGGCTCCGGGAGTGGTACGGATTTTACTTTAACCATTAGCCGCCTGGAACCAGAGGACTTCGC

GGTGTACTATTGTCTGCAGTGGAACTACTTCCCTTATACCTTTGGCCAGGGGACGAAAGTCGAGATTAAACGGACCGTGG
CGGCGCCGAGCGTGTTTATTTTTCCGCCGAGCGATGAACAGCTGAAAAGCGGCACCGCGAGCGTGGTGTGCCTGCTGAAC
AACTTTTATCCGCGTGAAGCGAAAGTGCAGTGGAAAGTGGATAACGCGCTGCAGAGCGGCAACAGCCAGGAAAGCGTGAC
CGAACAGGATAGCAAAGATAGCACCTATAGCCTGAGCAGCACCCTGACCCTGAGCAAAGCGGATTATGAAAAACATAAAG
TGTATGCGTGCGAAGTGACCCATCAGGGCCTGAGCAGCCCGGTGACCAAGAGCTTTAACCGTGGCGAATCTTAATCTAGA
TTAATTAAAGGAGAATTGAATATGAAATATCTTCTGCCGACTGCTGCGGCAGGCCTGTTATTGCTGGCGGCCGCTCCAGC
CATGGCTGAGGTACAGCTGCTTGAAAGCGGCGGTGGCCTGGTGCAACCGGGTGGGAGCCTGCGTCTGTCGTGCGCAGCCT
CCGGATTTACGTTCTCCAGCTACGCGATGAATTGGGTCCGTCAGGCTCCAGGTAAGGGTCTCGAGTGGGTCAGTCAAATC
AATCCTTCTGGTGGGAGCACGTATTATGCAGATAGCGTGAAGGGTCGCTTCACCATCTCCCGGGACAATTCGAAGAACAC
ACTGTATCTCCAAATGAACTCGCTTCGTGCTGAGGACACTGCCGTCTACTACTGTGTTGGTCATGAATGGGGTCAGGGTA
CACTAGTCACCGTGAGCTCGGCGTCTACCAAAGGCCCTTCGGTTTTTCCGCTGGCACCGTCTTCTAAAAGCACCAGTGGT
GGCACGGCTGCGCTGGGTTGCCTGGTTAAAGATTATTTTCCAGAGCCCGTGACAGTGTCGTGGAACTCAGGCGCGCTGAC
CAGCGGTGTGCATACCTTTCCCGCCGTTCTCCAGTCTTCCGGTTTATATTCTTTAAGCAGCGTGGTGACCGTGCCGTCTA
GCAGCTTGGGCACCCAGACCTATATTTGCAACGTGAATCATAAACCGAGCAACACCAAAGTGGATAAAAAGGTCGAACCG
AAGTCTAGC

Variant 4

ATGAAATACCTATTGCCTACGGCAGCCGCTGGATTGTTATTACTCGCGGCCCAGCCGGCCATGGCGGAGATCGTGCTGAC
CCAATCTCCGGGCACACTGAGCTTGTCTCCGGGCGAACGTGCGACCCTTAGCTGCAGAGCCAGCCAGTCGGTGTCCAGCT
CGTCTCTTGATTGGTATCAACAGAAACCAGGTCAAGCCCCGCGTTTATTAATTTACGGAGCATCTAGCCGCGCGACTGGC
GTGCCTGATCGTTTTTCCGGCTCCGGGAGTGGTACGGATTTTACTTTAACCATTAGCCGCCTGGAACCAGAGGACTTCGC
GGTGTACTATTGTCTGCAGTGGAACTACTTCCCTTATACCTTTGGCCAGGGGACGAAAGTCGAGATTAAACGGACTGTGG
CCGCACCTAGTGTTTTTATTTTTCCCCCTAGTGACGAACAGTTGAAGAGTGGTACTGCGAGTGTCGTGTGCCTGCTGAAT
AACTTTTACCCGCGTGAAGCGAAGGTACAGTGGAAAGTGGACAACGCGTTACAGTCGGGAAACTCTCAGGAATCCGTTAC
GGAACAGGATAGCAAAGATAGCACCTATAGCTTAAGCAGCACCCTGACACTGAGCAAGGCAGATTATGAAAAGCATAAGG
TTTATGCGTGCGAGGTTACCCACCAGGGCCTGAGCTCGCCGGTTACCAAGAGCTTTAACCGCGGTGAATCTTAGTCTAGA
TTAATTAAAGGAGAATTGAATATGAAATATCTTCTGCCGACTGCTGCGGCAGGCCTGCTGCTGCTGGCGGCCGCTCCAGC
CATGGCTGAGGTACAGCTGCTTGAAAGCGGCGGTGGCCTGGTGCAACCGGGTGGGAGCCTGCGTCTGTCGTGCGCAGCCT
CCGGATTTACGTTCTCCAGCTACGCGATGAATTGGGTCCGTCAGGCTCCAGGTAAGGGTCTCGAGTGGGTCAGTCAAATC
AATCCTTCTGGTGGGAGCACGTATTATGCAGATAGCGTGAAGGGTCGCTTCACCATCTCCCGGGACAATTCGAAGAACAC
ACTGTATCTCCAAATGAACTCGCTTCGTGCTGAGGACACTGCCGTCTACTACTGTGTTGGTCATGAATGGGGTCAGGGTA
CACTAGTCACCGTGAGCTCGGCGTCTACCAAAGGCCCTTCGGTTTTTCCGCTGGCACCGTCTTCTAAAAGCACCAGTGGT
GGCACGGCTGCGCTGGGTTGCCTGGTTAAAGATTATTTTCCAGAGCCCGTGACAGTGTCGTGGAACTCAGGCGCGCTGAC
CAGCGGTGTGCATACCTTTCCCGCCGTTCTCCAGTCTTCCGGTTTATATTCTTTAAGCAGCGTGGTGACCGTGCCGTCTA
GCAGCTTGGGCACCCAGACCTATATTTGCAACGTGAATCATAAACCGAGCAACACCAAAGTGGATAAAAAGGTCGAACCG
AAGTCTAGC

Variant 5

ATGAAATACCTATTGCCTACGGCAGCCGCTGGATTGTTATTACTCGCGGCCCAGCCGGCCATGGCGGAGATCGTGCTGAC
CCAATCTCCGGGCACACTGAGCTTGTCTCCGGGCGAACGTGCGACCCTTAGCTGCAGAGCCAGCCAGTCGGTGTCCAGCT
CGTCTCTTGATTGGTATCAACAGAAACCAGGTCAAGCCCCGCGTTTATTAATTTACGGAGCATCTAGCCGCGCGACTGGC
GTGCCTGATCGTTTTTCCGGCTCCGGGAGTGGTACGGATTTTACTTTAACCATTAGCCGCCTGGAACCAGAGGACTTCGC
GGTGTACTATTGTCTGCAGTGGAACTACTTCCCTTATACCTTTGGCCAGGGGACGAAAGTCGAGATTAAACGGACTGTGG
CCGCACCTAGTGTTTTTATTTTTCCCCCTAGTGACGAACAGTTGAAGAGTGGTACTGCGAGTGTCGTGTGCCTGCTGAAT
AACTTTTACCCGCGTGAAGCGAAGGTACAGTGGAAAGTGGACAACGCGTTACAGTCGGGAAACTCTCAGGAATCCGTTAC
GGAACAGGATAGCAAAGATAGCACCTATAGCTTAAGCAGCACCCTGACACTGAGCAAGGCAGATTATGAAAAGCATAAGG
TTTATGCGTGCGAGGTTACCCACCAGGGCCTGAGCTCGCCGGTTACCAAGAGCTTTAACCGCGGTGAATCTTAGTCTAGA
TTAATTAAAGGAGAATTGAATATGAAATATCTTCTGCCGACTGCTGCGGCAGGCCTGTTATTGCTGGCGGCCGCTCCAGC
CATGGCTGAGGTACAGCTGCTTGAAAGCGGCGGTGGCCTGGTGCAACCGGGTGGGAGCCTGCGTCTGTCGTGCGCAGCCT
CCGGATTTACGTTCTCCAGCTACGCGATGAATTGGGTCCGTCAGGCTCCAGGTAAGGGTCTCGAGTGGGTCAGTCAAATC
AATCCTTCTGGTGGGAGCACGTATTATGCAGATAGCGTGAAGGGTCGCTTCACCATCTCCCGGGACAATTCGAAGAACAC
ACTGTATCTCCAAATGAACTCGCTTCGTGCTGAGGACACTGCCGTCTACTACTGTGTTGGTCATGAATGGGGTCAGGGTA
CACTAGTCACCGTGAGCTCGGCGAGCACCAAAGGCCCGAGCGTGTTTCCGCTGGCGCCGAGCAGCAAAAGCACCAGCGGC
GGCACCGCGGCGCTGGGCTGCCTGGTGAAAGATTATTTTCCGGAACCGGTGACCGTGAGCTGGAACAGCGGCGCGCTGAC
CAGCGGCGTGCATACCTTTCCGGCGGTGCTGCAGAGCAGCGGCCTGTATAGCCTGAGCAGCGTGGTGACCGTGCCGAGCA
GCAGCCTGGGCACCCAGACCTATATTTGCAACGTGAACCATAAACCGAGCAACACCAAAGTGGATAAAAAAGTGGAACCG
AAAAGCAGC

Supplementary figure S2. The nucleotide sequences of the parent genes and the variants. (a) The nucleotide sequence of the parent genes sFab and Fab0. Differences between the parent genes are marked with red. As in the supplementary figure S1, different segments are separated by the vertical broken lines and numbered in the same manner. (b) The nucleotide sequences of the variants.

Supplementary methods. Detailed description of the cloning of the variants

For the production of variants 1, 2 and 3, parent gene Fab0 in pEB32x vector and sFab in pUC57 vector were first amplified in the reactions containing 1 x Phusion HF reaction buffer (Thermo Scientific), 200 µM dNTP mix (Thermo Scientific), 1 U Phusion Hot Start II polymerase (Thermo Scientific), 10 ng template and 0.5 µM primers. For the amplification of pEB32x-Fab0, primer pair TS3 (5' – CCCAATACGCAAACCGCCTCT – 3') and pAKrev (5' – CGCCATTTTTCACTTCACAG – 3') was used. For the amplification of pUC57-sFab, primer pair TS3 and WO536 (5' – GTAAAACGACGGCCAGT – 3') was used. Thermal cycling conditions were initial denaturation 98 °C for 30 s, denaturation 98 °C for 5 s, annealing 66 °C (Fab0) and 68 °C (sFab) for 20 s, extension 72 °C for 35 s and final extension 72 °C for 5 min. PCR cycle was repeated 25 (pEB32x-Fab0) and 30 (pUC57-sFab) times. After PCR purification, amplified Fab0 gene was digested with SfiI (Thermo Scientific). Digestion reaction was inactivated and purified with PCR purification kit. Subsequently, SfiI digested Fab0 was further digested in three separate reactions with (1) SexAI, with (2) SexAI and MscI and with (3) MscI and PacI. After digestion, target fragments (1) 1273 bp^{V1}, (2) 1094 bp^{V2} and 131 bp^{V2}, (3) 734 bp^{V3} and 310 bp^{V3} were obtained by gel extraction. Amplified sFab gene was digested in the same manner as Fab0 and (1) 131 bp^{V1}, (2) 179 bp^{V2} and (3) 360 bp^{V3} target fragments were extracted from the gel. The variants 1, 2 and 3 were then formed by mixing appropriate target fragments (all the fragments having the same superscript) and SfiI digested pEB32x vector together in equal molar ratios and ligating them with T4 DNA Ligase (Thermo Scientific) in three o/n reactions.

For the production of variants 4 and 5, the parent genes Fab0 and sFab in PEB32x and pUC57 vectors were digested with SfiI, PacI and XhoI in two separate reactions: (1) XhoI and SfiI and (2) XhoI and PacI. Target fragments (1) 885^{V5} bp and (2) 5486 bp^{V4} were obtained from Fab0 digestion. Target fragments (1) 519^{V5} bp and (2) 215^{V4} bp were obtained from sFab digestion. The variant 4 was formed by mixing the appropriate target fragments (all the fragments having the same superscript) of Fab0 and sFab with T4 DNA Ligase in molar ratio of 1:5, respectively. Variant 5

was formed using "PCR-after-ligation" method. The appropriate target fragments 885 bp (Fab0) and 519 bp (sFab) and SfiI digested pEB32x vector were first ligated with T4 DNA ligase. Molar ratio was 1:1:1. Ligation reaction was amplified in the reaction containing 1 x Phusion HF reaction buffer (Thermo Scientific), 200 µM dNTP mix (Thermo Scientific), 1 U Phusion Hot Start II polymerase (Thermo Scientific) and 0,5 µM primers. Primer pair WO375 (5' – TCACACAGGAAACAGCTATGAC – 3') and pAKrev was used. Thermal cycling condition was initial denaturation 98 °C for 30 s, denaturation 98 °C for 5 s, annealing 65 °C for 20 s, extension 72 °C for 31 s and final extension 72 °C for 5 min. PCR cycle was repeated 30 times. PCR reaction was purified with PCR purification kit and subsequently digested with SfiI. Reaction was purified with PCR purification kit and ligated to SfiI digested pEB32x vector with T4 DNA ligase. Molar ratio of pEB32x and digested PCR product was 1:3, respectively. All variants were transformed into *Escherichia coli* by electroporation (1.25 kV, 25 µFd, 200 Ω) and plated on LA plates (0.5 % glucose + 25 µg/ml chloramphenicol + 10 µg/ml tetracycline). Minipreps were produced and validity of the variants was confirmed by sequencing. After confirmation of validity, all variants were subcloned into pLK04 and pAK400 vectors using T4 DNA ligase and SfiI sites. Molar ratio of vectors and inserts were 1:3. Again, the validity was confirmed by sequencing.
